# User Experience, Engagement, and Popularity in Mental Health Apps: Secondary Analysis of App Analytics and Expert App Reviews

**DOI:** 10.2196/30766

**Published:** 2022-01-31

**Authors:** Benjamin T Kaveladze, Akash R Wasil, John B Bunyi, Veronica Ramirez, Stephen M Schueller

**Affiliations:** 1 Department of Psychological Science University of California Irvine, CA United States; 2 Department of Psychology University of Pennsylvania Philadelphia, CA United States; 3 Department of Informatics University of California Irvine, CA United States

**Keywords:** mental health apps, engagement, user experience, digital mental health, user retention

## Abstract

**Background:**

User experience and engagement are critical elements of mental health apps’ abilities to support users. However, work examining the relationships among user experience, engagement, and popularity has been limited. Understanding how user experience relates to engagement with and popularity of mental health apps can demonstrate the relationship between subjective and objective measures of app use. In turn, this may inform efforts to develop more effective and appealing mental health apps and ensure that they reach wide audiences.

**Objective:**

We aimed to examine the relationship among subjective measures of user experience, objective measures of popularity, and engagement in mental health apps.

**Methods:**

We conducted a preregistered secondary data analysis in a sample of 56 mental health apps. To measure user experience, we used expert ratings on the Mobile App Rating Scale (MARS) and consumer ratings from the Apple App Store and Google Play. To measure engagement, we acquired estimates of monthly active users (MAU) and user retention. To measure app popularity, we used download count, total app revenue, and MAU again.

**Results:**

MARS total score was moderately positively correlated with app-level revenue (Kendall rank [T]=0.30, *P*=.002), MAU (T=0.39, *P*<.001), and downloads (T=0.41, *P*<.001). However, the MARS total score and each of its subscales (Engagement, Functionality, Aesthetics, and Information) showed extremely small correlations with user retention 1, 7, and 30 days after downloading. Furthermore, the total MARS score only correlated with app store rating at T=0.12, which, at *P*=.20, did not meet our threshold for significance.

**Conclusions:**

More popular mental health apps receive better ratings of user experience than less popular ones. However, user experience does not predict sustained engagement with mental health apps. Thus, mental health app developers and evaluators need to better understand user experience and engagement, as well as to define sustained engagement, what leads to it, and how to create products that achieve it. This understanding might be supported by better collaboration between industry and academic teams to advance a science of engagement.

## Introduction

### Background

An increasing number of mental health apps are available to consumers, with estimates that 10,000 to 20,000 mental health apps currently exist [1,2]. Evidence suggests that these apps can help address various mental health concerns such as stress, depression, and anxiety. Even unguided apps intended for self-management can lead to reliable, albeit small, benefits [3], particularly for people with lower symptom severity [4]. The biggest challenge facing these apps, especially when provided in unguided, direct-to-consumer models, is engagement. Engagement with most mental health apps is abysmal—estimates suggest that most publicly available mental health apps for depression and anxiety have zero or near-zero active users [5]. This study aims to better understand engagement with mental health apps from the vantage points of user experience and popularity.

### Previous Work on Mental Health App Engagement

Previous research has examined mental health app engagement in multiple ways [6]. A scoping review of concepts and components of engagement used in the digital health literature emphasized that engagement is a multifaceted concept with behavioral, cognitive, and affective components [7]. Although self-report engagement measures aim to capture some of these components [8], in this paper we focus on the behavioral component using analytic data, which tracks users’ actual usage of apps (eg, number of downloads or average time per use). However, analytic data can also be used to determine different conceptualizations of engagement, and we consider some of these approaches below.

One approach to studying engagement is to quantify user retention: the proportion of users who continue to use an app over a certain period. Estimates suggest that approximately 4% of users who download a mental health app continue using it after 15 days, and 3% continue after 30 days [9]. Early efforts have also identified some factors that predict retention, such as therapeutic persuasiveness and therapeutic alliance [10]. Retention is a useful metric of engagement because it considers sustained use rather than only initial adoption (downloading an app).

Another perspective on engagement focuses on monthly active users (MAU): the number of people who use an app in a given month. Most mental health apps have nearly no active users, while a few apps have millions of active users [5]. This trend of vastly unequal distributions of users across similar apps seems to be true not only for mental health apps but also for apps focused on physical fitness and mood-tracking [11]. Given these extreme differences in MAU, efforts to understand how highly popular apps differ from unpopular apps have been a priority. Importantly, an app’s MAU reflects two distinct components: (1) the number of people who downloaded the app (which reflects an app’s popularity and marketing success) and (2) retention (which may reflect content and features within the app). Because retention data are often difficult to obtain, investigators recently proposed an alternative “stickiness” metric, defined as the number of monthly active users per normalized total downloads [12]. Interestingly, some of the most downloaded apps do not appear to be particularly sticky, and some of the stickiest apps are not the most downloaded [12].

There have also been efforts to improve engagement with digital mental health interventions. While this work is in its early stages, some promising strategies include incorporating human-centered design principles [13], branding digital mental health interventions in ways that appeal to specific subgroups of users [14], sending reminders and “digital triggers” [15], and incorporating human support [16].

In summary, current research has examined engagement from multiple perspectives, several efforts to improve engagement are underway, and research on engagement is still in its infancy. One important next step involves understanding why some apps are more engaging or more popular than others. Such work could inform efforts to increase engagement by highlighting specific content, features, characteristics, or development strategies that may contribute to engagement.

### Characteristics of User Experience

User experience refers to the holistic experience of using a product such as a mobile app. It is shaped by an app’s content, its functionality, and its look and feel. Similar to conceptualizations of engagement, it also encompasses affective, behavioral, and cognitive reactions and includes emotional, hedonic, and aesthetic variables [17]. User experience can be understood through various methods including expert or heuristic evaluations, user interviews, and user reviews [18]. As user experience is a multifaceted and complex concept, different methods of understanding user experience have relative strengths and weaknesses.

For mobile mental health apps, the most widely used measure of user experience is the mobile app rating scale (MARS) [19]. It has been used in various evaluations of health apps, including mindfulness apps [20] and pain-management apps [21]. The MARS evaluates mobile health app quality along dimensions of engagement, functionality, aesthetics, and information quality. The engagement scale assesses how interactive and interesting the app is, the functionality scale assesses the app’s functioning and ease of use, the aesthetics scale assesses overall visual appeal and stylistic consistency, and the information subscale assesses the quality of the content. Averaged together, the 4 subscales form the MARS total score, which measures overall app quality. Typically, the MARS is used by a trained evaluator with expertise in some facet of mobile health apps such as technical or clinical expertise or lived experience with the health condition. In this way, the MARS can be thought of as a form of heuristic evaluation where experts score various components of the app using validated metrics. The MARS’s construct validity was established by confirming its factor structure, and its concurrent validity was established by relating it to another app quality assessment tool [22]; however, more research is needed to determine the MARS’s other psychometric properties, such as its predictive validity.

Another way to understand the user experience of mental health apps is to ask consumers, either directly through user experience interviews or indirectly by analyzing consumer reviews posted to app stores [23]. Interviews can provide in-depth information but are labor-intensive and may not accurately reflect user behavior. App store reviews are easily accessible and plentiful for popular apps, but review-writers’ perspectives may not be representative of most of an app’s users. Studies that directly ask consumers about their experiences and those that leverage app store reviews can provide converging evidence of characteristics that are important for consumers such as a positive framing or simplicity [24,25]. A review of studies of mental health app user experience identified six themes among consumers’ perceptions of apps: helpfulness, enhancements, technical issues, ease of use, satisfaction, and perceived issues [26]. Additionally, a study examining over 13,000 reviews of 106 mental health apps noted that user interface and user-friendliness were two of the most common aspects commented on by users and that poor usability was often noted as a reason for abandoning apps [23]. Although these themes align with some aspects of the MARS subscales, such as functionality, they also tend to correspond to more general perceptions of quality or specific improvements or deficits.

To summarize, methods for evaluating user experience for mobile health apps have been refined over the past years and produced useful insights into consumer preferences. Nonetheless, better understanding user experiences is critical because, ultimately, mental health apps are beneficial only insofar as users meaningfully engage with them.

### This Study

This study aimed to identify associations between mental health app user experience and metrics of app popularity and engagement. We first hypothesized that more popular apps, in terms of app-level revenue, monthly active users, and downloads, would have higher user experience ratings. Second, we hypothesized that apps’ levels of engagement, functionality, and aesthetic appeal would predict user retention more strongly than their informational quality. Third, we hypothesized that app store ratings would be correlated with user experience ratings.

## Methods

### Design and Material

We obtained MARS scores from One Mind PsyberGuide, a nonprofit organization that provides structured reviews of mental health apps [27]. One Mind PsyberGuide reviews mental health apps on multiple metrics including user experience as defined by the MARS. Three reviewers with training on MARS administration—2 PhD-level reviewers, each with extensive experience in user experience and mental health app reviews, and 1 individual with lived experience of mental health issues—completed each MARS review. These 3 ratings were averaged to produce the MARS scores provided on One Mind PsyberGuide and used in our analyses. Overall, we had access to MARS ratings from 91 mental health apps, including total score and all 4 MARS subscales. The ratings were completed between March 2020 and December 2020.

We obtained analytic data from Apptopia, a company that aggregates data on various metrics of mobile app usage and popularity [28]. This analytic data included app-level data on MAU (ie, the number of users who opened the app at least once in the past 30 days), daily revenue (in US$), daily downloads, app store rating (1-5, ratings were obtained from Google Play and Apple App Store and the mean across stores was used when data from both stores were available), and user retention variables corresponding to 1, 2, 3, 4, 5, 6, 7, 14, and 30 days after downloading the app (with values 0-100 corresponding to the percentage of people who opened the app *n* days after downloading it). The MAU, daily revenue, and daily downloads variables had daily values for each day between February 8, 2020, and February 8, 2021, which is a 1-year period that overlaps with that of the MARS ratings. We transformed these daily values to a single value per app, computed as the variable’s mean across all of the days of the month in which that app’s MARS review was completed. We performed all analyses using these month-averaged data, rather than the daily values. Because mean values as a measure of central tendency are susceptible to influence by outliers or skewness of the distribution, we also computed the variable’s median values across all of the days of the month and report all analyses using these month median data in Multimedia Appendix 1. The app store rating variable and each retention variable had only one value in our data set. For app store rating, the value corresponded to the average of all app store ratings in the 365 days preceding February 8, 2021. Each retention variable’s value reflected the average of retention values across every day in January 2021.

### Exclusion Criteria

We chose to exclude several apps from analyses owing to missing data. First, we excluded apps that lacked Apptopia data for at least 1 day in the month as missing data may have created a bias toward inflated monthly average values. Among the 91 apps with MARS rating data provided by One Mind PsyberGuide, 56 had Apptopia data for every day of the month that the MARS review was completed, and 54 of those 56 had data for user retention and app store ratings. In total, 18 of the 91 apps with MARS rating data had no Apptopia data whatsoever. Thus, for some analyses we include 56 apps and for others we include 54 apps.

### Data Availability

Our hypotheses and analysis plan were preregistered and are available on the internet, as well as the data sets we used for analyses (in addition to data on additional variables for each app) [29]. We have also provided the output from the main and sensitivity analyses in Multimedia Appendix 1. Owing to Apptopia’s data-sharing policy, we have provided the Apptopia data separately with the app names deleted and the apps presented in random order.

### Analyses

Statistical analyses were performed using the stats package in R (R Core Team, 2020). For all analyses, statistical significance was set at a preregistered threshold *P*<.05; however, we have reported exact *P* values unless they were <.001. Data manipulation and figure creation were conducted in R using the tidyverse family of packages [30] and sjPlot [31].

For our first hypothesis—that more popular apps, in terms of app-level revenue, MAU, and downloads, would have higher MARS scores—we determined the Kendall rank (T) correlation coefficients (3 in total) between the MARS total score and revenue, MAU, and downloads. We used the Kendall rank correlation coefficient rather than the Pearson correlation coefficient because rank-order correlation is not overly impacted by the presence of extreme outliers (and we knew there were several such outliers in the revenue, MAU, and downloads variables in our data set) and is therefore more consistent with our research question. Nonetheless, this skewness remains important to consider when interpreting our results.

For our second hypothesis—that the MARS engagement, functionality, and aesthetic subscales would predict user retention more strongly than the information subscale—we chose to calculate Kendall rank correlation coefficients (12 in total) between each MARS subscale of interest (Engagement, Functionality, Aesthetics, and Information) and user retention, as measured by the percentage of users who downloaded the app who opened it 1, 7, and 30 days after download.

For our third hypothesis—that app store ratings would be correlated with the MARS total score—we chose to calculate the Kendall rank correlation coefficient between the MARS total score and app store rating.

## Results

### Results Overview

**Figure 1:**
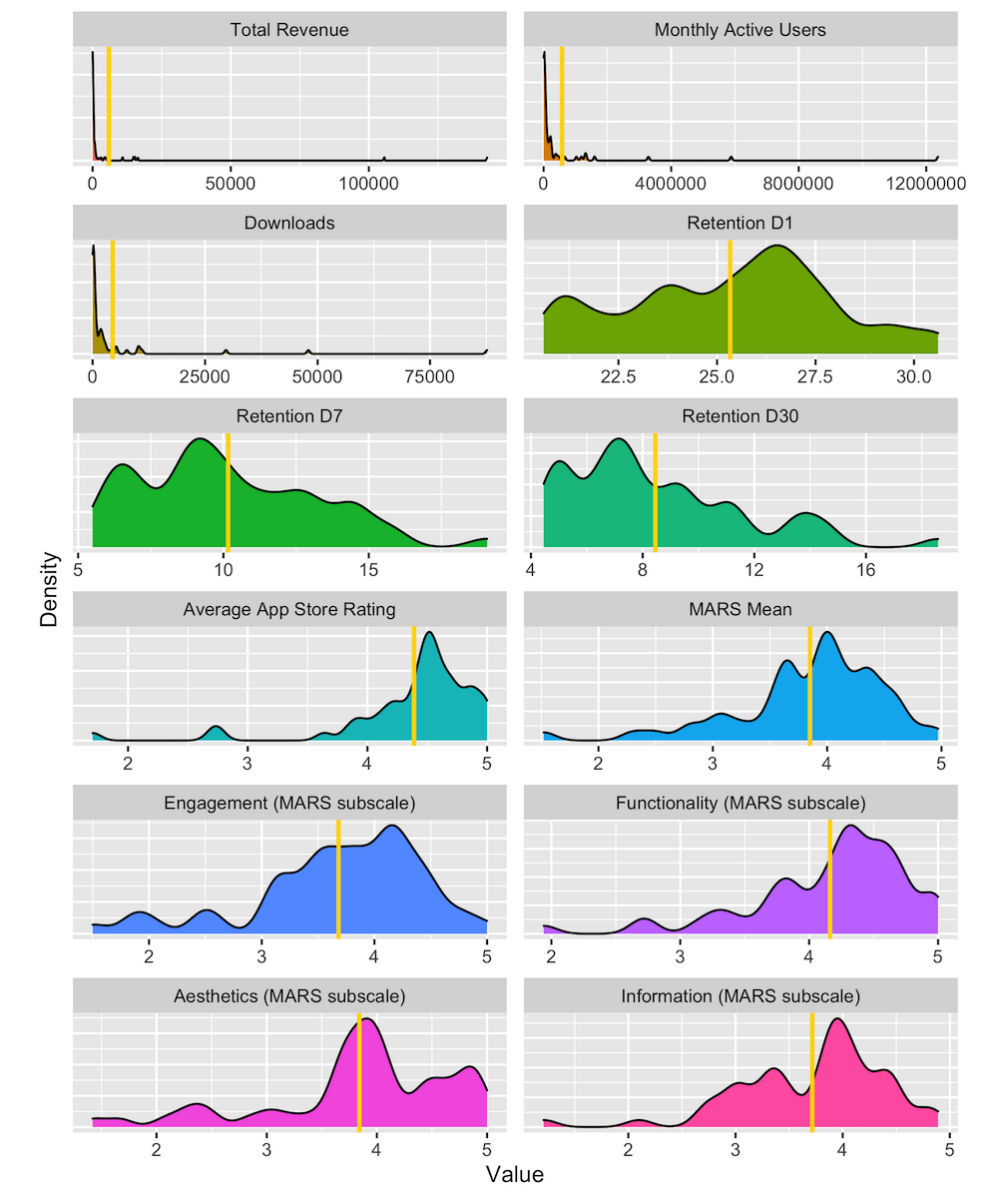
Frequency distributions for all variables (n=54 or n=56) included in our analyses. Variable mean values are shown with gold vertical lines. MARS: Mobile App Rating Scale.

**Figure 2:**
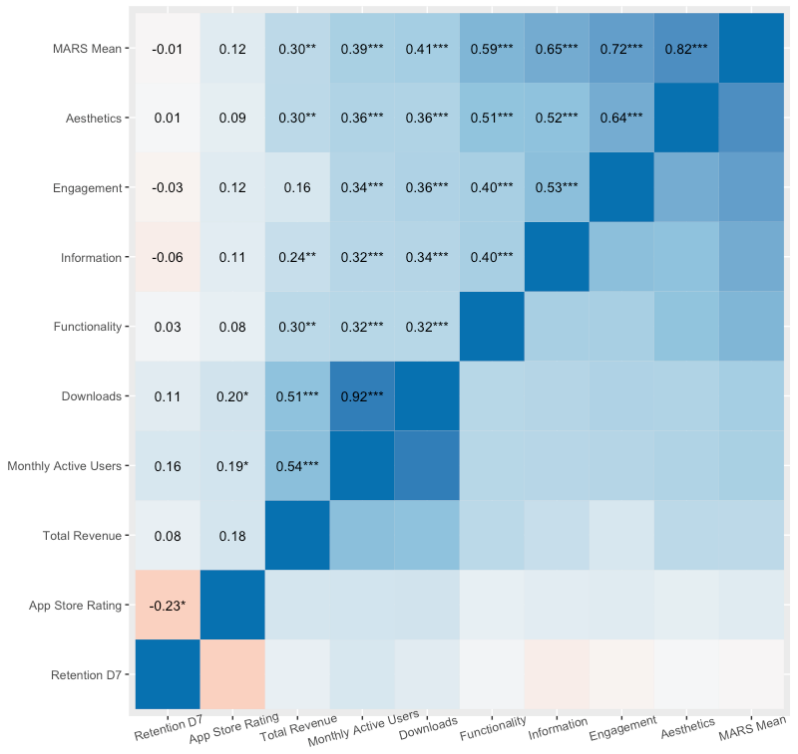
Kendall rank correlation coefficients between all variables (n=54 or n=56). Pairwise deletion was used to deal with missing data. *P<.05. **P<.01. ***P<.001. MARS: Mobile App Rating Scale.

Figure 1 shows variable distributions and mean values, and Figure 2 illustrates Kendall rank correlation coefficients across variables of interest. MARS total scores tended to be high (mean 3.85, SD 0.65), as did the MARS subscales for engagement (mean 3.68, SD 0.76), functionality (mean 4.16, SD 0.61), aesthetics (mean 3.84, SD 0.84), and information (mean 3.72, SD 0.69). App store ratings were also high (mean 4.39, SD 0.59). Revenue, MAU, and downloads were highly skewed owing to a few extremely popular outliers.

### Distribution of App Usage

The distribution of monthly active users among the 56 mental health apps we examined was highly skewed (mean 578,645, SD 1,856,468, median 48,676, range 0-12,373,122). Among all the apps in our data set, the 3 most popular apps accounted for 66.6% of MAUs, and the 10 most popular apps accounted for 90% of MAUs [11]. As noted in the analyses section, we account for these distributions’ skewness by using Kendall rank correlations in our analyses.

### Associations Between MARS Scores and App Popularity, User Retention, and App Store Ratings

**Figure 3:**
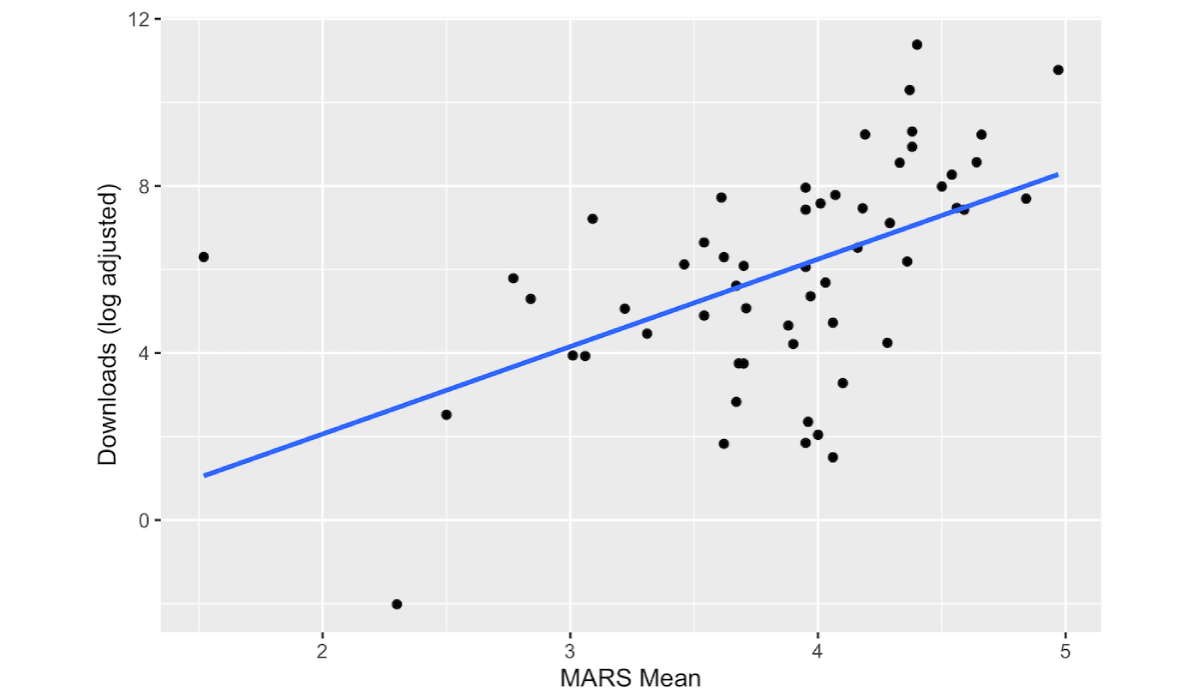
The association between Mobile App Rating Scale (MARS) Mean scores and log-adjusted downloads for mobile mental health apps, Kendall T=0.41, *P*<.001, n=56.

In our sample of 56 mental health apps, Kendall rank correlation analyses revealed that the MARS total score was moderately positively correlated with app-level revenue (T=0.30, *P*=.002), MAU (T=0.39, *P*<.001), and downloads (T=0.41, *P*<.001; Figure 3). Conversely, in our sample of 54 mental health apps, the MARS total score and its subscales (ie, Engagement, Functionality, Aesthetics, and Information) showed minor associations with user retention 1, 7, and 30 days after downloading (T=–0.10 to 0.17) and none of these associations met our threshold for significance (*P*=.93 to .07). Lastly, the MARS total score was also extremely weakly correlated with app store ratings (T=0.12), which did not meet our threshold for significance (*P*=.20).

### Sensitivity Analysis

The daily values that were averaged to form the monthly average values for the MAU, daily revenue, and daily downloads variables were not normally distributed for many apps, although they also did not have extreme outliers. As sensitivity analysis, we reran the analyses using the median of the MAU, daily revenue, and daily downloads variables instead of the mean. The results from the two approaches were nearly identical, and we have included the output from the analyses using median values in Multimedia Appendix 1.

## Discussion

### Principal Findings

Our findings support one of our three hypotheses. Specifically, we found that user experience scores were related to several app popularity metrics: downloads, revenue, and monthly active users. However, none of the MARS subscales were predictive of user retention. Therefore, user experience, at least as defined by the MARS, is a fairly good indicator of how many people might start to engage with, or adopt, an app but may be less informative about users’ sustained engagement with the app. Further, the lack of a correlation between user experience (as measured by the MARS total score) and app store ratings suggests that app store ratings are not a broadly useful measure of user experience.

### Relationship Between Popularity Metrics and User Experience

The moderate rank-order correlations we observed between popularity or revenue and user experience suggest that higher scores on user experience, as rated by individual observers, are characteristic of more popular apps. Importantly, however, this relationship does not appear linear: a few apps are responsible for nearly all users, suggesting that marginal improvements to popularity may not be sufficient to retain users. Instead, reaching a “popularity threshold” may be necessary, with apps below that threshold being unlikely to gain many users.

These findings suggest that industry teams, rather than academic ones, may be best-suited to create highly engaging and popular products. To date, nearly all of the most engaging digital mental health interventions have been developed by industry teams, rather than academic teams [12,32]. It is plausible that differences in funding sources (eg, federal grants vs flexible capital), incentive structures (eg, priorities on publishing vs marketing), timelines (eg, multi-year studies vs rapid testing of prototypes), and other factors may give industry teams a competitive advantage in developing highly engaging products. As an example, Headspace and Calm, the two most popular mental health and wellness apps, each raised over US $140 million in funding from venture capital firms [33]. Furthermore, industry teams are often diverse and interdisciplinary and charged not only with developing an engaging product but also marketing and financing that product. Aspects of business models and marketing may play an important role in people’s likelihood to adopt or sustain use of a given app. For example, word of mouth is a common way that people learn about mental health apps [1]. Payment models also impact both adoption and sustainment; people prefer free apps [1] but dislike “freemium pricing” [34]. Some mental health apps have also used celebrity advertising, such as LeBron James for Calm or Michael Phelps for Talkspace. This paper focused on aspects of the apps themselves rather than these other aspects of the business models and advertising, but those aspects are worth exploring in further work.

### The Need to Better Advance a Science of Engagement

A common presumption is that mental health apps require sustained engagement for users to experience their intended benefits. Taking a similar approach to the National Institute of Mental Health Research Domain Criteria framework, Graham et al [35] propose that engagement is a critical mechanism of action for mental health apps. In their conceptualization, engagement can be separated into elements that speak to design targets such as usefulness, usability, and satisfaction, as well as use metrics, such as those used in our analyses. Our findings suggest, however, that use metrics might speak to different aspects of engagement, and that more work needs to unpack elements that lead people to adopt, use, and explore technologies. This is logically similar to efforts in implementation science frameworks to map key implementation outcomes including adoption, appropriateness, and sustainment, and consistent with work that has mapped those outcomes onto different variables relevant to technology-enabled services [36].

As can be seen in Figure 2, while our measure of initial engagement—ie, downloads—was predicted by several variables, our measure of sustained engagement—ie, retention—was not. While retention was quite low in general, it still varied considerably across apps (retention 7 days after download ranged from 5.5% to 19.1%). Our study echoes previous research suggesting that retention is particularly challenging to understand [9]. One reason that retention may be so challenging to predict using the statistical approaches typical in behavioral sciences is that on the user level, retention’s distribution is highly skewed: most users do not engage with a given app more than once or twice but some engage much more often; hence, mean retention values do not represent most users’ experiences. More in-depth forms of analysis such as user interviews and longitudinal analyses may be required to understand patterns in mental health app user retention. These qualitative approaches can go beyond just how many people use or stop using an app to explore richer questions regarding users’ journeys with an app. In turn, these data can help to identify critical aspects of the user experience.

It is also worth noting that user engagement, even retention, is likely a heterogeneous concept. Even among users who are considered to be retained, patterns of use might differ, including among different dimensions such as frequency (as in consistent vs bursty use), intensity (as in moderate or super users [37]), time (as in circadian patterns in use [38]), or type (as in using clinically meaningful app features [39]). These dimensions similarly characterize other types of complex behavior such as exercise (ie, the Frequency, Intensity, Time, and Type model). For some users, decreased use over time could be a sign that the app improved their well-being or that the user completed the app’s intervention as intended. Therefore, although much has been made of the poor rates of long-term sustainment in mental health apps [9-11], some users likely experience “happy abandonment,” wherein a lack of sustained use suggests they received what they needed. Unfortunately, given that our user retention information was obtained from app-level analytic data, we were not able to determine individual-level characteristics of retention and engagement. However, future work could help determine the degree to which app engagement patterns are shaped by characteristics of the app, such as user experience or app features, and characteristics of the different ways that people use digital health products.

Another reason that retention might be a heterogeneous concept is that mental health apps vary in their intended user journeys. Some apps might be designed for people to use them every day, whereas others might be designed for more emergent yet infrequent situations. Therefore, in addition to individual-level characteristics of retention and engagement, it is also worth noting that retention might have app-level characteristics; as such, retention may not allow apples-to-apples comparisons of apps. Again, because our data were obtained from an analytics platform rather than the apps themselves, we were not able to conduct more nuanced analyses of retention; however, efforts that could combine and synthesize engagement data across platforms [40] could help investigate these questions among others.

Given that achieving sustained engagement is so difficult for mental health apps, an alternative strategy involves circumventing the challenge of long-term engagement altogether by creating digital interventions that are designed to confer benefit rapidly. An example comes from the growing literature on digital single-session interventions, which are designed to produce benefits after just one sitting [41-43]. These interventions attempt to reimagine how to support users’ mental health in ways that differ from typical therapist-client interactions but might hold greater appeal and utility.

### Limitations

There are several limitations to consider in this work. First, the set of 56 apps observed in this study (for which One Mind PsyberGuide chose to complete MARS reviews and for which engagement data were available from Apptopia) are not representative of the full array of available mental health apps, with a likely bias toward more popular apps and those designed for English-speaking audiences. Nonetheless, these apps represented a fairly wide range of values across all variables. Second, the MARS may not be an ideal measure of user experience. Although many of the elements in the MARS address user engagement, it is often conceptualized as an overall measure of app quality, rather than solely user experience [19]. Furthermore, some aspects of user experience within apps might not be captured by the MARS, such as gamification principles [44]. Third, because the study is cross-sectional and observational, we are unable to infer causality. Many of the observed relationships between variables are likely bidirectional; for example, better user experience likely causes apps to become more popular, but apps that are more popular also gain the resources to improve their user experience design. Fourth, apps differed in the time distances between their respective MARS review dates and the dates for which their rating and retention data were available, although all MARS ratings occurred during the 1-year period for which analytic data were obtained. Lastly, as the aim of this paper was to understand how aspects of user experience relate to engagement and popularity, we do not know if using these mental health apps actually helps people to achieve their goals in using these apps or to derive clinical benefits. In this study, we did not have access to analytic data on user outcomes, but such data would be a strength of a solution that facilitates better collaboration with developers for analysis and evaluation purposes.

### Future Directions

Although this analysis observes rank-order trends, it does not explain why a few apps, such as Calm and Headspace, are exponentially more popular than others. Future research can explore the complex combination of factors, such as marketing dollars and market trends, which could explain these few apps’ outsized popularity. Such research might also explore the optimal conditions for making influential and effective apps. For example, industry teams tend to create more popular and engaging apps than academic teams do; however, solutions to user engagement problems plaguing these apps might be best pursued by rigorous research combining quantitative, qualitative, and experimental approaches [45]. Thus, research could examine if collaborations between academic and industry teams may be particularly fruitful in creating evidence-based and highly scalable interventions. Finally, given low retention among mental health apps, future work should explore innovative intervention strategies by which apps can support mental health in ways that appeal to users.

As one resource for exploring these future directions and other ideas, we encourage researchers to explore the publicly available data sets that we used to conduct our analyses [29], which contain data on more apps and more variables (including credibility, intervention target, intervention approach, app price, and average time spent per session) than those examined in this study.

### Conclusions

We found that popular mental health apps—as defined by their number of downloads, revenue, and number of monthly active users—tend to be rated as having a better user experience than less popular apps. We also noted that user retention metrics are not well-predicted by other app-level metrics. We encourage further collaboration between industry and academic teams to better advance a science of engagement and to create more effective and appealing mental health apps.

## Appendix

Multimedia Appendix 1 Preregistered analysis and sensitivity analysis output.

## Abbreviations

MARS: Mobile App Rating Scale
MAU: monthly active users
