# Supplementary material for: User Experience, Engagement, and Popularity in Mental Health Apps: Secondary Analysis of App Analytics and Expert App Reviews
Source: JMIR Hum Factors. 2022 Jan 31;9(1):e30766. doi: 10.2196/30766 (PMC8844980; doi:10.2196/30766)

UX and MARS paper analysis output

```r
#User Experience, Engagement, and Popularity in Mental Health Apps: Secondary Analysis of App Analytics and Exper
t App Reviews
#Benjamin T. Kaveladze, Akash R. Wasil, John B, Bunyi, Veronica Ramirez, & Stephen M. Schueller
#### DUE TO APPTOPIA DATA SHARING RULES, WE CANNOT PROVIDE THE DATA TO REPLICATE THESE
#### ANALYSES.
```

variable distributions

```r
for_figure%>% ggplot(aes(x=value,fill=name))+
  geom_density(show.legend = F,adjust=1/2)+
  geom_vline(data = for_figure, mapping = aes(xintercept = m),
             size=1,color='gold') +
  facet_wrap(vars(name),scales='free',ncol=2)+ggtitle('Distributions of variables (n=54-56)')+
  theme( axis.ticks.y = element_blank(),axis.text.y = element_blank())
```

```
## Warning: Removed 8 rows containing non-finite values (stat_density).
```

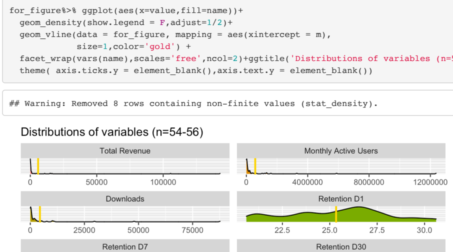


```r
#ggsave('Variable distributions.png', plot = last_plot(),height=8)

df_app %>%
  rename('Engagement (MARS subscale)'=Engagement,
         'Functionality (MARS subscale)'=Functionality,
         'Aesthetics (MARS subscale)'=Aesthetics,
         'Information (MARS subscale)'=Information,
         'Average App Store Rating'='Average Rating')%>%
  select(all_of(vars_2select))%>%
  pivot_longer(everything())  %>% group_by(name) %>%
  add_count(n2=!is.na(value)) %>% filter(n2==T) %>%
  summarize(mean(value,na.rm=T),med=median(value,na.rm=T),
            sd=sd(value,na.rm=T),n) %>% slice(1)
```

```
## `summarise()` has grouped output by 'name'. You can override using the `.groups` argument.
```

```
## # A tibble: 12 x 5
## # Groups:   name [12]
##    name                          `mean(value, na.rm = …      med       sd     n
##    <chr>                                          <dbl>    <dbl>    <dbl> <int>
##  1 Aesthetics (MARS subscale)                      3.84     3.92  8.44e-1    56
##  2 Average App Store Rating                        4.39     4.51  5.92e-1    54
##  3 Downloads                                    4455.     448.    1.37e+4    56
##  4 Engagement (MARS subscale)                      3.68     3.82  7.61e-1    56
##  5 Functionality (MARS subscale)                   4.16     4.31  6.14e-1    56
##  6 Information (MARS subscale)                     3.72     3.90  6.85e-1    56
##  7 MARS Mean                                       3.85     3.96  6.50e-1    56
##  8 Monthly Active Users                       578645.   48676.    1.86e+6    56
##  9 Retention D1                                   25.3     25.6   2.63e+0    54
## 10 Retention D30                                   8.45     7.53  3.16e+0    54
## 11 Retention D7                                   10.2      9.73  3.07e+0    54
## 12 Total Revenue                                5875.      46.4   2.35e+4    56
```

Pre-registered analyses

```r
#H1: Larger-scale apps, in terms of app-level revenue, monthly active users, and
#downloads, will tend to have higher MARS Means.
cor.test(df_app$`MARS Mean`,df_app$`Total Revenue`,method='kendall')
```

```
##
##  Kendall's rank correlation tau
##
## data:  df_app$`MARS Mean` and df_app$`Total Revenue`
## z = 3.1359, p-value = 0.001713
## alternative hypothesis: true tau is not equal to 0
## sample estimates:
##       tau
## 0.2964674
```

```r
df_app %>% ggplot(aes(`MARS Mean`,log(`Total Revenue`)))+
  geom_point()+geom_smooth(method = 'lm',se=F) +
  ylab('Total Revenue (log adjusted)')+
  ggtitle('MARS Mean Predicting App Revenue',"Kendall's Tau= 0.29")
```

```
## `geom_smooth()` using formula 'y ~ x'
```

```
## Warning: Removed 15 rows containing non-finite values (stat_smooth).
```

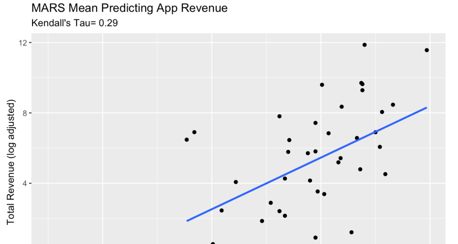


```r
cor.test(df_app$`MARS Mean`,df_app$MAU,method='kendall')
```

```
##
##  Kendall's rank correlation tau
##
## data:  df_app$`MARS Mean` and df_app$MAU
## z = 4.2562, p-value = 0.00002079
## alternative hypothesis: true tau is not equal to 0
## sample estimates:
##       tau
## 0.3924411
```

```r
df_app %>% ggplot(aes(`MARS Mean`,log(MAU)))+
  geom_point()+geom_smooth(method = 'lm',se=F) +
  ylab('Monthly Active Users (log adjusted)')+ggtitle('MARS Mean Predicting Monthly Active Users')
```

```
## `geom_smooth()` using formula 'y ~ x'
```

```
## Warning: Removed 1 rows containing non-finite values (stat_smooth).
```

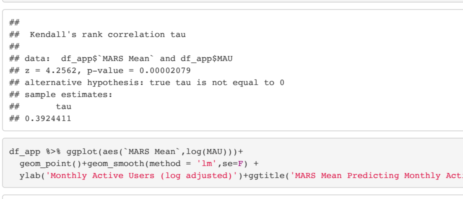


```r
cor.test(df_app$`MARS Mean`,df_app$Downloads,method='kendall')
```

```
##
##  Kendall's rank correlation tau
##
## data:  df_app$`MARS Mean` and df_app$Downloads
## z = 4.3976, p-value = 0.00001094
## alternative hypothesis: true tau is not equal to 0
## sample estimates:
##      tau
## 0.405479
```

```r
df_app %>% ggplot(aes(`MARS Mean`,log(Downloads)))+
  geom_point()+geom_smooth(method = 'lm',se=F)+
  ylab('Downloads (log adjusted)')+
  ggtitle('MARS Mean Predicting Downloads',"Kendall's Tau= 0.41, p< 0.001,n=56")
```

```
## `geom_smooth()` using formula 'y ~ x'
```

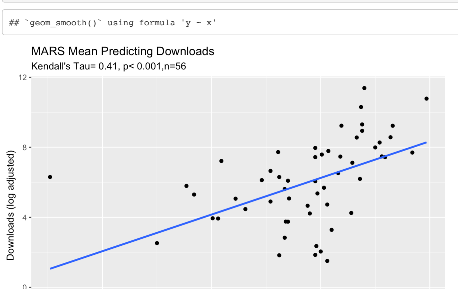


```r
#note that Downloads and MAU are correlated 0.99, so its redundant to
#include both in the same paper. Revenue is correlated with them ~.45

#H2:The MARS's Engagement, Functionality, and Aesthetics subscales will predict
#user retention more strongly than the Information subscale
cor.test(df_app$Engagement,df_app$`Retention D1`,method='kendall')
```

```
##
##  Kendall's rank correlation tau
##
## data:  df_app$Engagement and df_app$`Retention D1`
## z = 1.195, p-value = 0.2321
## alternative hypothesis: true tau is not equal to 0
## sample estimates:
##       tau
## 0.1130402
```

```r
plot(df_app$Engagement,df_app$`Retention D1`)
```

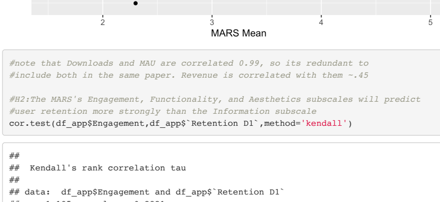


```r
cor.test(df_app$Functionality,df_app$`Retention D1`,method='kendall')
```

```
##
##  Kendall's rank correlation tau
##
## data:  df_app$Functionality and df_app$`Retention D1`
## z = 1.7951, p-value = 0.07263
## alternative hypothesis: true tau is not equal to 0
## sample estimates:
##       tau
## 0.1715306
```

```r
cor.test(df_app$Aesthetics,df_app$`Retention D1`,method='kendall')
```

```
##
##  Kendall's rank correlation tau
##
## data:  df_app$Aesthetics and df_app$`Retention D1`
## z = 1.6747, p-value = 0.09399
## alternative hypothesis: true tau is not equal to 0
## sample estimates:
##       tau
## 0.1595137
```

```r
cor.test(df_app$Information,df_app$`Retention D1`,method='kendall')
```

```
##
##  Kendall's rank correlation tau
##
## data:  df_app$Information and df_app$`Retention D1`
## z = 0.58962, p-value = 0.5554
## alternative hypothesis: true tau is not equal to 0
## sample estimates:
##        tau
## 0.05547802
```

```r
cor.test(df_app$`MARS Mean`,df_app$`Retention D1`,method='kendall')
```

```
##
##  Kendall's rank correlation tau
##
## data:  df_app$`MARS Mean` and df_app$`Retention D1`
## z = 1.6942, p-value = 0.09023
## alternative hypothesis: true tau is not equal to 0
## sample estimates:
##       tau
## 0.1592992
```

```r
cor.test(df_app$Engagement,df_app$`Retention D7`,method='kendall')
```

```
##
##  Kendall's rank correlation tau
##
## data:  df_app$Engagement and df_app$`Retention D7`
## z = -0.26887, p-value = 0.788
## alternative hypothesis: true tau is not equal to 0
## sample estimates:
##         tau
## -0.02543404
```

```r
cor.test(df_app$Functionality,df_app$`Retention D7`,method='kendall')
```

```
##
##  Kendall's rank correlation tau
##
## data:  df_app$Functionality and df_app$`Retention D7`
## z = 0.27675, p-value = 0.782
## alternative hypothesis: true tau is not equal to 0
## sample estimates:
##        tau
## 0.02644429
```

```r
cor.test(df_app$Aesthetics,df_app$`Retention D7`,method='kendall')
```

```
##
##  Kendall's rank correlation tau
##
## data:  df_app$Aesthetics and df_app$`Retention D7`
## z = 0.089717, p-value = 0.9285
## alternative hypothesis: true tau is not equal to 0
## sample estimates:
##         tau
## 0.008545374
```

```r
cor.test(df_app$Information,df_app$`Retention D7`,method='kendall')
```

```
##
##  Kendall's rank correlation tau
##
## data:  df_app$Information and df_app$`Retention D7`
## z = -0.6344, p-value = 0.5258
## alternative hypothesis: true tau is not equal to 0
## sample estimates:
##         tau
## -0.05969154
```

```r
cor.test(df_app$`MARS Mean`,df_app$`Retention D7`,method='kendall')
```

```
##
##  Kendall's rank correlation tau
##
## data:  df_app$`MARS Mean` and df_app$`Retention D7`
## z = -0.097025, p-value = 0.9227
## alternative hypothesis: true tau is not equal to 0
## sample estimates:
##          tau
## -0.009122863
```

```r
cor.test(df_app$Engagement,df_app$`Retention D30`,method='kendall')
```

```
##
##  Kendall's rank correlation tau
##
## data:  df_app$Engagement and df_app$`Retention D30`
## z = -0.60494, p-value = 0.5452
## alternative hypothesis: true tau is not equal to 0
## sample estimates:
##        tau
## -0.0572066
```

```r
cor.test(df_app$Functionality,df_app$`Retention D30`,method='kendall')
```

```
##
##  Kendall's rank correlation tau
##
## data:  df_app$Functionality and df_app$`Retention D30`
## z = -0.32162, p-value = 0.7477
## alternative hypothesis: true tau is not equal to 0
## sample estimates:
##         tau
## -0.03072182
```

```r
cor.test(df_app$Aesthetics,df_app$`Retention D30`,method='kendall')
```

```
##
##  Kendall's rank correlation tau
##
## data:  df_app$Aesthetics and df_app$`Retention D30`
## z = -0.35138, p-value = 0.7253
## alternative hypothesis: true tau is not equal to 0
## sample estimates:
##         tau
## -0.03345769
```

```r
cor.test(df_app$Information,df_app$`Retention D30`,method='kendall')
```

```
##
##  Kendall's rank correlation tau
##
## data:  df_app$Information and df_app$`Retention D30`
## z = -1.1046, p-value = 0.2693
## alternative hypothesis: true tau is not equal to 0
## sample estimates:
##        tau
## -0.1038972
```

```r
cor.test(df_app$`MARS Mean`,df_app$`Retention D30`,method='kendall')
```

```
##
##  Kendall's rank correlation tau
##
## data:  df_app$`MARS Mean` and df_app$`Retention D30`
## z = -0.55228, p-value = 0.5808
## alternative hypothesis: true tau is not equal to 0
## sample estimates:
##       tau
## -0.051912
```

```r
# H3: Google Play store and Apple app store ratings will be correlated with the MARS mean.
cor.test(df_app$`Average Rating`,df_app$`MARS Mean`,method='kendall')
```

```
##
##  Kendall's rank correlation tau
##
## data:  df_app$`Average Rating` and df_app$`MARS Mean`
## z = 1.2769, p-value = 0.2016
## alternative hypothesis: true tau is not equal to 0
## sample estimates:
##       tau
## 0.1205925
```

```r
with(df_app,plot(`MARS Mean`,`Average Rating`))
```

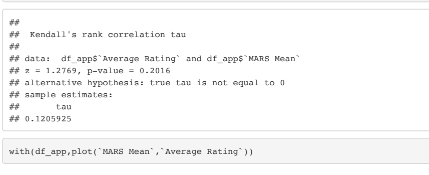

Additional analyses

```r
#avg session length and quality
cor.test(df_app$avg_session_seconds,df_app$`MARS Mean`,method='kendall')
```

```
## Warning in cor.test.default(df_app$avg_session_seconds, df_app$`MARS Mean`, :
## Cannot compute exact p-value with ties
```

```
##
##  Kendall's rank correlation tau
##
## data:  df_app$avg_session_seconds and df_app$`MARS Mean`
## z = 0.4535, p-value = 0.6502
## alternative hypothesis: true tau is not equal to 0
## sample estimates:
##        tau
## 0.04543434
```

```r
#Downloads and MARS subscales
cor.test(df_app$Downloads,df_app$Aesthetics,method='kendall')
```

```
##
##  Kendall's rank correlation tau
##
## data:  df_app$Downloads and df_app$Aesthetics
## z = 3.8528, p-value = 0.0001168
## alternative hypothesis: true tau is not equal to 0
## sample estimates:
##       tau
## 0.3598501
```

```r
cor.test(df_app$Downloads,df_app$Information,method='kendall')
```

```
##
##  Kendall's rank correlation tau
##
## data:  df_app$Downloads and df_app$Information
## z = 3.6341, p-value = 0.0002789
## alternative hypothesis: true tau is not equal to 0
## sample estimates:
##       tau
## 0.3352938
```

```r
cor.test(df_app$Downloads,df_app$Engagement,method='kendall')
```

```
##
##  Kendall's rank correlation tau
##
## data:  df_app$Downloads and df_app$Engagement
## z = 3.9264, p-value = 0.00008624
## alternative hypothesis: true tau is not equal to 0
## sample estimates:
##       tau
## 0.3640726
```

```r
cor.test(df_app$Downloads,df_app$Functionality,method='kendall')
```

```
##
##  Kendall's rank correlation tau
##
## data:  df_app$Downloads and df_app$Functionality
## z = 3.4427, p-value = 0.0005758
## alternative hypothesis: true tau is not equal to 0
## sample estimates:
##       tau
## 0.3221356
```

```r
library(sjPlot)
```

```
## Registered S3 methods overwritten by 'parameters':
##   method                           from
##   as.double.parameters_kurtosis    datawizard
##   as.double.parameters_skewness    datawizard
##   as.double.parameters_smoothness  datawizard
##   as.numeric.parameters_kurtosis   datawizard
##   as.numeric.parameters_skewness   datawizard
##   as.numeric.parameters_smoothness datawizard
##   print.parameters_distribution    datawizard
##   print.parameters_kurtosis        datawizard
##   print.parameters_skewness        datawizard
##   summary.parameters_kurtosis      datawizard
##   summary.parameters_skewness      datawizard
```

```
## Install package "strengejacke" from GitHub (`devtools::install_github("strengejacke/strengejacke")`) to load a
ll sj-packages at once!
```

```r
d <- df_app %>%
  select(`MARS Mean`,Engagement:Information,credibility,`Average Rating`,
         `Retention D7`:avg_session_seconds)
d_smaller <- d %>%
  select(-c(credibility,DAU,`Total Time Spent In App`,`Retention D30`,
          avg_session_seconds,Sessions,ARPU,`Retention D14`)) %>%
  rename('Monthly Active Users'='MAU','App Store Rating'='Average Rating')
sjp.corr(d_smaller,title = 'Kendall Tau Correlations (n=54-56)',
         decimals=2,corr.method = "kendall",na.deletion = 'pairwise')+
  theme(axis.text.x = element_text(angle=15,hjust=0.5))
```

```
## Warning: 'sjp.corr' is deprecated. Please use 'correlation::correlation()' and
## its related plot()-method.
```

```
## Computing correlation using kendall-method with pairwise-deletion...
```

```
## Warning: Removed 55 rows containing missing values (geom_text).
```

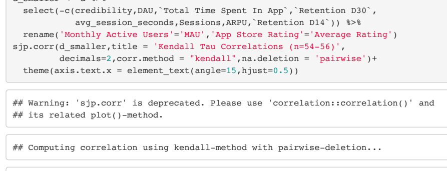


```r
#ggsave('corz.png', plot = last_plot(), width = 8, height = 8, dpi = 100)
```

Checking for normality of daily values and re-running analyses with median instead of mean

```r
full_for_normality_test <- full %>% group_by(app_id) %>%
  mutate(month_match=case_when(month(apptopia_date)==month(`Date of Rating`)-1,TRUE~0)) %>%
  filter(month_match==1) %>%
  mutate(apptopia_count=n()) %>%
  filter(days_in_month(`Date of Rating`)==apptopia_count) %>%
  filter(DAU>0,MAU>0,Downloads>0,Sessions>0,`Total Time Spent In App`>0,
         `Total Revenue`>0,ARPU>0)
#p-values for each variable
full_for_normality_test %>% group_by(app_id) %>% select(-app_name,-`App Name`) %>%
  summarize(across(DAU:`Total Revenue`,~ shapiro.test(.)$p.value)) #isn't very normal
```

```
## # A tibble: 38 x 7
##    app_id       DAU       MAU Downloads  Sessions `Total Time Spe… `Total Revenue`
##    <chr>      <dbl>     <dbl>     <dbl>     <dbl>            <dbl>           <dbl>
##  1 a1     0.0000168 0.0403    0.0838    0.00335            0.800         0.0180
##  2 a10    0.584     0.0785    0.562     0.635              0.912         0.257
##  3 a11    0.00203   0.00202   0.0171    0.0125             0.0506        0.357
##  4 a12    0.0000246 0.190     0.0444    0.0000450          0.496         0.00857
##  5 a13    0.117     0.000132  0.000221  0.0110             0.688         0.0299
##  6 a17    0.0306    0.0614    0.326     0.00847            0.733         0.440
##  7 a19    0.0262    0.102     0.0727    0.00587            0.313         0.000140
##  8 a2     0.00113   0.0217    0.0781    0.00109            0.0424        0.00915
##  9 a20    0.0495    0.0151    0.775     0.727              0.133         0.775
## 10 a25    0.0340    0.0000101 0.000463  0.0334             0.0205        0.0144
## # … with 28 more rows
```

```r
full_short_median <- Rating_Database %>% left_join(
  full %>% group_by(app_id) %>%
    mutate(month_match=case_when(month(apptopia_date)==month(`Date of Rating`)-1,TRUE~0)) %>%
    filter(month_match==1) %>%
    mutate(across(DAU:ARPU,~ median(.,na.rm=T)),
           apptopia_count=n(),`Store Name`=`Store Name`) %>%
    slice(1) %>%
    select(app_id,`Average Rating`:`Retention D30`, DAU:ARPU,avg_session_seconds,
           apptopia_count,`Store Name`) ,by='app_id') %>%
  mutate(price=as.numeric(price))

df_app_median <- full_short_median %>%
  filter(days_in_month(`Date of Rating`)==apptopia_count) %>%
  mutate(Sessions=case_when(Sessions==0&MAU!=0~NaN,TRUE~Sessions))

#H1: Larger-scale apps, in terms of app-level revenue, monthly active users, and
#downloads, will tend to have higher MARS Means.
cor.test(df_app_median$`MARS Mean`,df_app_median$`Total Revenue`,method='kendall')
```

```
##
##  Kendall's rank correlation tau
##
## data:  df_app_median$`MARS Mean` and df_app_median$`Total Revenue`
## z = 3.032, p-value = 0.00243
## alternative hypothesis: true tau is not equal to 0
## sample estimates:
##       tau
## 0.2904989
```

```r
df_app_median %>% ggplot(aes(`MARS Mean`,log(`Total Revenue`)))+
  geom_point()+geom_smooth(method = 'lm',se=F) +
  ylab('Total Revenue (log adjusted)')+
  ggtitle('MARS Mean Predicting App Revenue',"Kendall's Tau= 0.29")
```

```
## `geom_smooth()` using formula 'y ~ x'
```

```
## Warning: Removed 19 rows containing non-finite values (stat_smooth).
```

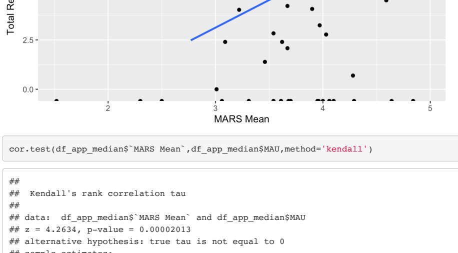


```r
cor.test(df_app_median$`MARS Mean`,df_app_median$MAU,method='kendall')
```

```
##
##  Kendall's rank correlation tau
##
## data:  df_app_median$`MARS Mean` and df_app_median$MAU
## z = 4.2634, p-value = 0.00002013
## alternative hypothesis: true tau is not equal to 0
## sample estimates:
##       tau
## 0.3932207
```

```r
df_app_median %>% ggplot(aes(`MARS Mean`,log(MAU)))+
  geom_point()+geom_smooth(method = 'lm',se=F) +
  ylab('Monthly Active Users (log adjusted)')+ggtitle('MARS Mean Predicting Monthly Active Users')
```

```
## `geom_smooth()` using formula 'y ~ x'
```

```
## Warning: Removed 2 rows containing non-finite values (stat_smooth).
```

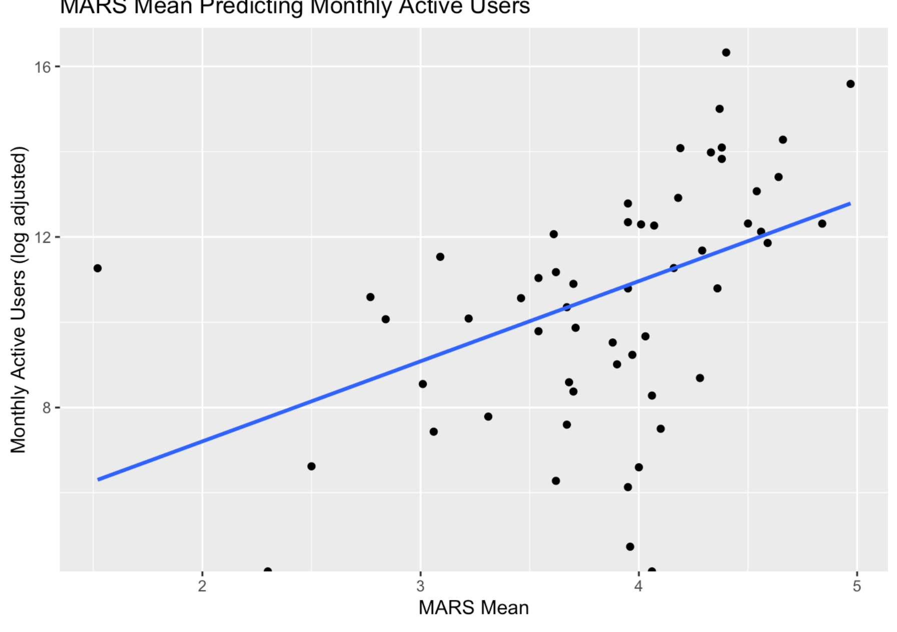


```
cor.test(df_app_median$`MARS Mean`,df_app_median$Downloads,method='kendall')
```

```
##
##  Kendall's rank correlation tau
##
## data:  df_app_median$`MARS Mean` and df_app_median$Downloads
## z = 4.4331, p-value = 0.00000929
## alternative hypothesis: true tau is not equal to 0
## sample estimates:
##       tau
## 0.4088712
```

```
df_app_median %>% ggplot(aes(`MARS Mean`,log(Downloads)))+
  geom_point()+geom_smooth(method = 'lm',se=F)+
  ylab('Downloads (log adjusted)')+
  ggtitle('MARS Mean Predicting Downloads',"Kendall's Tau= 0.41, p< 0.001,n=56")
```

```
## `geom_smooth()` using formula 'y ~ x'
```

```
## Warning: Removed 1 rows containing non-finite values (stat_smooth).
```

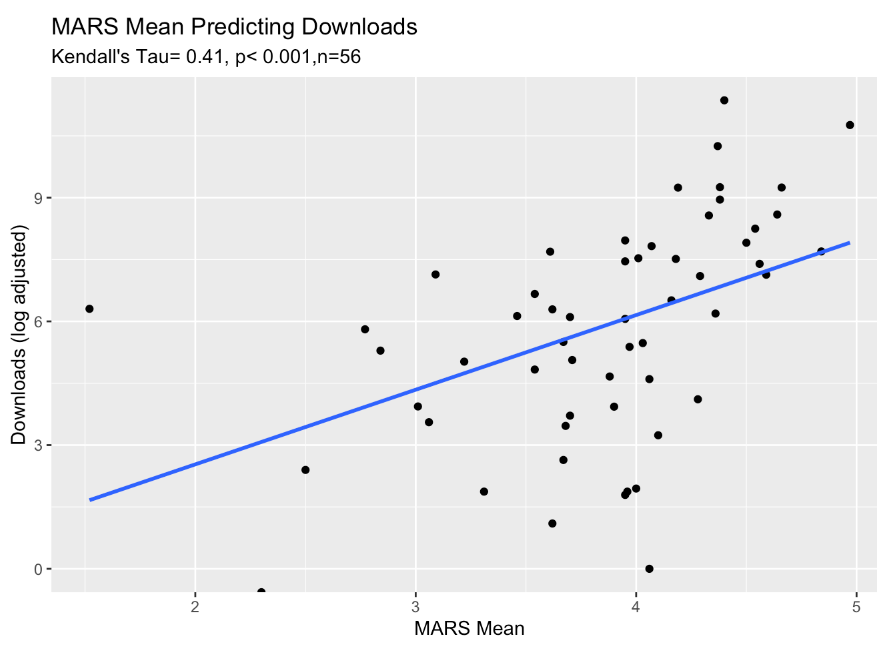


```
#note that Downloads and MAU are correlated 0.99, so its redundant to
#include both in the same paper. Revenue is correlated with them ~.45


#H2:The MARS's Engagement, Functionality, and Aesthetics subscales will predict
#user retention more strongly than the Information subscale
cor.test(df_app_median$Engagement,df_app_median$`Retention D1`,method='kendall')
```

```
##
##  Kendall's rank correlation tau
##
## data:  df_app_median$Engagement and df_app_median$`Retention D1`
## z = 1.195, p-value = 0.2321
## alternative hypothesis: true tau is not equal to 0
## sample estimates:
##       tau
## 0.1130402
```

```
plot(df_app_median$Engagement,df_app_median$`Retention D1`)
```

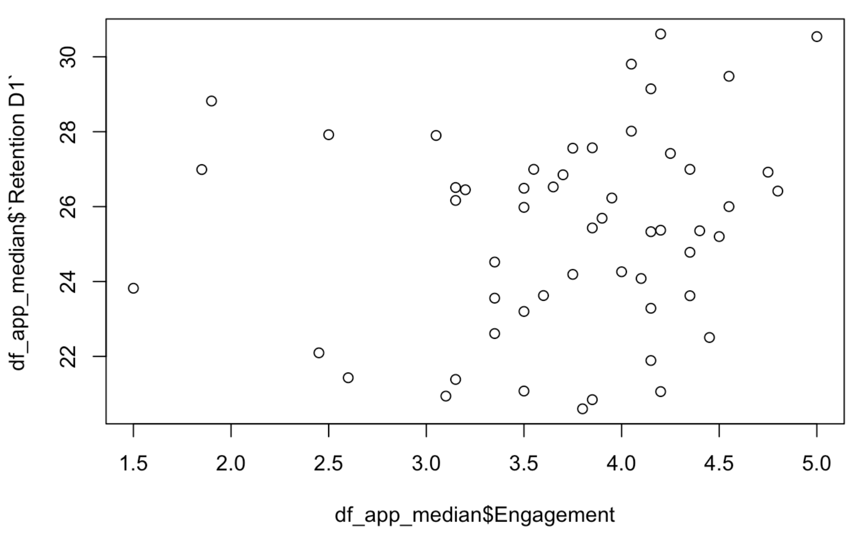


```
cor.test(df_app_median$Functionality,df_app_median$`Retention D1`,method='kendall')
```

```
##
##  Kendall's rank correlation tau
##
## data:  df_app_median$Functionality and df_app_median$`Retention D1`
## z = 1.7951, p-value = 0.07263
## alternative hypothesis: true tau is not equal to 0
## sample estimates:
##       tau
## 0.1715306
```

```
cor.test(df_app_median$Aesthetics,df_app_median$`Retention D1`,method='kendall')
```

```
##
##  Kendall's rank correlation tau
##
## data:  df_app_median$Aesthetics and df_app_median$`Retention D1`
## z = 1.6747, p-value = 0.09399
## alternative hypothesis: true tau is not equal to 0
## sample estimates:
##       tau
## 0.1595137
```

```
cor.test(df_app_median$Information,df_app_median$`Retention D1`,method='kendall')
```

```
##
##  Kendall's rank correlation tau
##
## data:  df_app_median$Information and df_app_median$`Retention D1`
## z = 0.58962, p-value = 0.5554
## alternative hypothesis: true tau is not equal to 0
## sample estimates:
##        tau
## 0.05547802
```

```
cor.test(df_app_median$`MARS Mean`,df_app_median$`Retention D1`,method='kendall')
```

```
##
##  Kendall's rank correlation tau
##
## data:  df_app_median$`MARS Mean` and df_app_median$`Retention D1`
## z = 1.6942, p-value = 0.09023
## alternative hypothesis: true tau is not equal to 0
## sample estimates:
##       tau
## 0.1592992
```

```
cor.test(df_app_median$Engagement,df_app_median$`Retention D7`,method='kendall')
```

```
##
##  Kendall's rank correlation tau
##
## data:  df_app_median$Engagement and df_app_median$`Retention D7`
## z = -0.26887, p-value = 0.788
## alternative hypothesis: true tau is not equal to 0
## sample estimates:
##         tau
## -0.02543404
```

```
cor.test(df_app_median$Functionality,df_app_median$`Retention D7`,method='kendall')
```

```
##
##  Kendall's rank correlation tau
##
## data:  df_app_median$Functionality and df_app_median$`Retention D7`
## z = 0.27675, p-value = 0.782
## alternative hypothesis: true tau is not equal to 0
## sample estimates:
##        tau
## 0.02644429
```

```
cor.test(df_app_median$Aesthetics,df_app_median$`Retention D7`,method='kendall')
```

```
##
##  Kendall's rank correlation tau
##
## data:  df_app_median$Aesthetics and df_app_median$`Retention D7`
## z = 0.089717, p-value = 0.9285
## alternative hypothesis: true tau is not equal to 0
## sample estimates:
##         tau
## 0.008545374
```

```
cor.test(df_app_median$Information,df_app_median$`Retention D7`,method='kendall')
```

```
##
##  Kendall's rank correlation tau
##
## data:  df_app_median$Information and df_app_median$`Retention D7`
## z = -0.6344, p-value = 0.5258
## alternative hypothesis: true tau is not equal to 0
## sample estimates:
##         tau
## -0.05969154
```

```
cor.test(df_app_median$`MARS Mean`,df_app_median$`Retention D7`,method='kendall')
```

```
##
##  Kendall's rank correlation tau
##
## data:  df_app_median$`MARS Mean` and df_app_median$`Retention D7`
## z = -0.097025, p-value = 0.9227
## alternative hypothesis: true tau is not equal to 0
## sample estimates:
##          tau
## -0.009122863
```

```
cor.test(df_app_median$Engagement,df_app_median$`Retention D30`,method='kendall')
```

```
##
##  Kendall's rank correlation tau
##
## data:  df_app_median$Engagement and df_app_median$`Retention D30`
## z = -0.60494, p-value = 0.5452
## alternative hypothesis: true tau is not equal to 0
## sample estimates:
##        tau
## -0.0572066
```

```
cor.test(df_app_median$Functionality,df_app_median$`Retention D30`,method='kendall')
```

```
##
##  Kendall's rank correlation tau
##
## data:  df_app_median$Functionality and df_app_median$`Retention D30`
## z = -0.32162, p-value = 0.7477
## alternative hypothesis: true tau is not equal to 0
## sample estimates:
##         tau
## -0.03072182
```

```
cor.test(df_app_median$Aesthetics,df_app_median$`Retention D30`,method='kendall')
```

```
##
##  Kendall's rank correlation tau
##
## data:  df_app_median$Aesthetics and df_app_median$`Retention D30`
## z = -0.35138, p-value = 0.7253
## alternative hypothesis: true tau is not equal to 0
## sample estimates:
##         tau
## -0.03345769
```

```
cor.test(df_app_median$Information,df_app_median$`Retention D30`,method='kendall')
```

```
##
##  Kendall's rank correlation tau
##
## data:  df_app_median$Information and df_app_median$`Retention D30`
## z = -1.1046, p-value = 0.2693
## alternative hypothesis: true tau is not equal to 0
## sample estimates:
##        tau
## -0.1038972
```

```
cor.test(df_app_median$`MARS Mean`,df_app_median$`Retention D30`,method='kendall')
```

```
##
##  Kendall's rank correlation tau
##
## data:  df_app_median$`MARS Mean` and df_app_median$`Retention D30`
## z = -0.55228, p-value = 0.5808
## alternative hypothesis: true tau is not equal to 0
## sample estimates:
##       tau
## -0.051912
```

```
# H3: Google Play store and Apple app store ratings will be correlated with the MARS mean.
cor.test(df_app_median$`Average Rating`,df_app_median$`MARS Mean`,method='kendall')
```

```
##
##  Kendall's rank correlation tau
##
## data:  df_app_median$`Average Rating` and df_app_median$`MARS Mean`
## z = 1.2769, p-value = 0.2016
## alternative hypothesis: true tau is not equal to 0
## sample estimates:
##       tau
## 0.1205925
```

```
with(df_app_median,plot(`MARS Mean`,`Average Rating`))
```

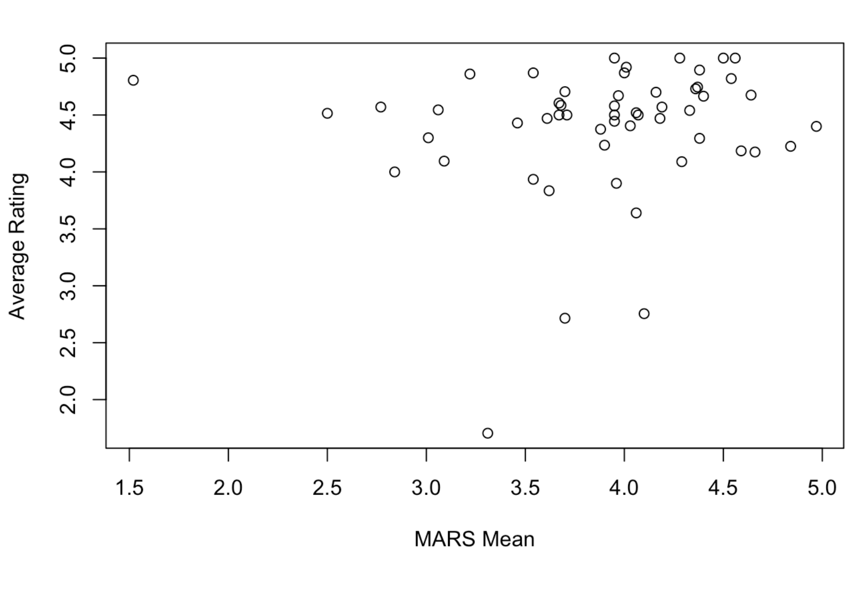

Supplement: Multimedia Appendix 1 [file humanfactors_v9i1e30766_app1.pdf]
